# Adaptive Step-Size Tuning Combined with Ternary Search Optimization for FCS-MPC Sensorless Speed Estimation of PMSMs

**DOI:** 10.3390/s26134118

**Published:** 2026-06-30

**Authors:** Zheng Dang, Fuguang Yang, Jiuhong Ruan, Maozhuang Tian, Dong Liu

**Affiliations:** 1 School of Rail Transportation, Shandong Jiaotong University, Jinan 250357, China; dang200172@163.com (Z.D.); 24221020@stu.sdjtu.edu.cn (M.T.); 25221029@stu.sdjtu.edu.cn (D.L.); 2 Key Laboratory of Rail Transit Safety Technology and Equipment, Shandong Province Transportation Industry, Jinan 250357, China

**Keywords:** sensorless speed estimation, PMSMs, FCS-MPC, adaptive step-size, ternary search

## Abstract

To address the slow transient convergence, low steady-state estimation accuracy and heavy computational complexity in finite control set model predictive control (FCS-MPC)-based sensorless speed estimation for permanent magnet synchronous motors (PMSMs). This paper proposes an adaptive step-size tuning strategy combined with ternary search optimization. Firstly, a cost function based on the flux linkage error is constructed, and the prediction step size of the electrical angle is adaptively adjusted according to the normalized flux linkage error. Specifically, a larger step size is adopted under transient conditions to accelerate convergence, whereas a smaller step size is adopted under steady-state operation to improve estimation precision. Secondly, a ternary search strategy is introduced to optimize the electrical-angle search process, reducing the number of candidate evaluations and the computational complexity. Finally, the effectiveness of the proposed method is validated through MATLAB/Simulink simulations and experiments on a PMSM test bench. Comparative results demonstrate that, under various operating conditions, the proposed method achieves faster transient convergence, higher steady-state estimation accuracy, and lower computational complexity than the conventional fixed step-size FCS-MPC speed estimation method.

## 1. Introduction

Permanent magnet synchronous motors have been extensively employed in industrial automation, electric vehicle drives, robotics, and high-performance advanced servo applications owing to their high efficiency, high power density, and superior dynamic performance [1,2]. In these applications, achieving high-precision and fast-response speed control is essential for ensuring system stability, improving energy efficiency, and enhancing dynamic performance [3]. As a typical multi-variable coupled nonlinear system, PMSMs are predominantly controlled using field-oriented control (FOC). By decoupling the current components within the synchronous rotating coordinate system, FOC enables PMSMs to achieve control characteristics similar to those of DC motors, making it the dominant strategy in modern motor drive systems [4]. However, the efficacy of FOC critically depends on accurate rotor position and speed information, which are typically obtained through mechanical sensors such as encoders or resolvers [5].

Although high-precision position signals can be provided by mechanical sensors, their use inevitably increases system cost, device volume, and installation complexity [6,7,8]. Furthermore, under adverse operating conditions, such as elevated temperature, strong vibration, and dusty environments, the reliability and stability of these sensors are significantly degraded, which further limits the comprehensive performance of the motor drive system [9]. To mitigate the dependence on physical position transducers, sensorless techniques are employed, wherein the rotor’s angular displacement and velocity are derived from intrinsic motor electrical variables. This approach reduces hardware expenditures and assembly intricacies while simultaneously enhancing the dependability and stability of the drive system [10].

Currently, prevailing sensorless estimation techniques for PMSMs are primarily composed of the model reference adaptive system (MRAS), sliding mode observer (SMO), extended Kalman filter (EKF), and MPC-based schemes [11]. MRAS is widely employed due to its straightforward configuration and computational efficiency; however, its efficacy is heavily dependent on the precise adjustment of proportional–integral (PI) parameters. Fixed parameter settings are often insufficient to cope with complex and time-varying operating conditions, thereby limiting the capacity for simultaneously achieving fast dynamic response and high steady-state estimation accuracy [12]. The SMO exhibits remarkable immunity to variations in system parameters and external noise, although its inherent oscillatory behavior may induce fluctuations in speed estimation, which can degrade control performance [13]. Stochastic filtering methods, such as the EKF, can provide high-precision state estimates even under measurement noise; nevertheless, their substantial computational complexity and reliance on accurate system models constrain their practical applicability, particularly in cost-sensitive drive platforms [14]. Consequently, achieving a balance between rapid transient response and accurate steady-state estimation remains challenging under complex operating conditions and strict real-time requirements [15].

Recently, considerable attention has been attracted by model predictive control (MPC) in electric motor drives owing to its outstanding dynamic characteristics and capability to address multi-objective control problems [16]. Among the various MPC approaches, the discrete nature of inverter switching states is exploited by FCS-MPC to explicitly determine the optimal control action within a finite candidate set based on a cost function and predicted system behavior [17,18]. This approach, which obviates the need for complex modulation stages and allows for the explicit incorporation of system constraints into the control process, demonstrates significant potential for motor control applications [19].

Despite the progress achieved by existing methods, several critical challenges are still faced by sensorless speed estimation based on predictive control. First, conventional fixed step-size strategies are often insufficient to reconcile the requirements of transient response and steady-state precision amid fluctuating operational states, which leads to degraded performance during either transient or steady-state operation [20,21]. Second, exhaustive evaluation of multiple discrete points is often required by traditional candidate angle search methods, resulting in high computational complexity. This issue is particularly severe in real-time control systems with high sampling rates and limited computational resources [22]. Consequently, the enhancement of search efficiency while maintaining high estimation accuracy, and the coordination between dynamic response and steady-state performance, remain key challenges for current sensorless speed estimation methods.

**Figure 1:**
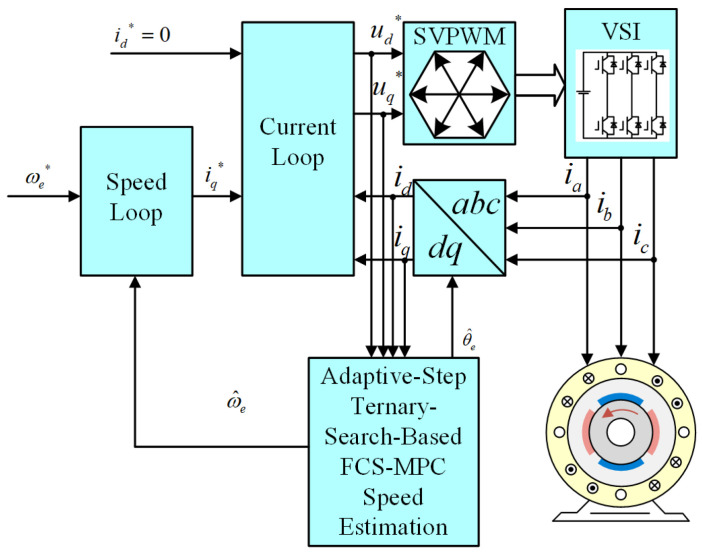
Overall block diagram of the sensorless speed estimation method based on adaptive step-size and ternary search-based FCS-MPC.

This study develops a variable step-size FCS-MPC-based sensorless speed estimation method to overcome the aforementioned challenges. In this approach, the electrical-angle estimation is formulated as a finite-set optimization problem by defining a cost function based on the flux linkage error for candidate angles. Simultaneously, to reduce the computational complexity of traditional exhaustive search methods, a ternary search strategy is employed to replace the multi-point enumeration method, thereby decreasing the number of evaluations and simplifying the algorithm. As illustrated in Figure 1, the proposed method first constructs a stator flux linkage model from measured voltage and current signals and establishes the corresponding cost function. The adaptive step-size mechanism is then integrated with the ternary search to efficiently estimate the electrical angle, and the rotor speed is finally determined based on the variation of the electrical angle between consecutive sampling intervals.

The main contributions of this study are summarized as follows:

(1) An adaptive step-size adjustment strategy for the electrical angle is proposed based on the norm of the flux linkage error. The step size is dynamically adjusted according to the estimated error, which achieves an improved balance between dynamic response and steady-state estimation accuracy.
(2) A ternary search strategy is introduced to optimize the electrical-angle search process. By replacing conventional point-by-point enumeration over a fixed candidate set, the total iteration count is substantially decreased by this method, which alleviates the computational complexity.
(3) The proposed method is verified via MATLAB 2024a/Simulink simulations and experiments on a PMSM platform. Compared with the conventional fixed step-size FCS-MPC, the results show that the method improves transient performance and steady-state estimation accuracy.

The remainder of this paper is organized as follows. Section 2 develops the mathematical model of the PMSM. Section 3 introduces the conventional fixed step-size FCS-MPC speed estimation method. Section 4 presents the proposed adaptive step-size and ternary search-based FCS-MPC speed estimation method. Section 5 reports simulation and experimental results with detailed analysis. Finally, Section 6 concludes the study.

## 2. Mathematical Model of the PMSM

To enable the formulation and evaluation of control strategies, a mathematical representation of the PMSM is developed under the following assumptions [23]:

(1) The air-gap magnetic flux is assumed to be sinusoidally distributed.
(2) Magnetic saturation, hysteresis, and eddy-current effects are neglected.
(3) The stator windings are symmetrical, and the motor parameters are constant.
(4) The permanent magnet flux linkage is assumed to be constant and unaffected by temperature variations.

Under these assumptions, the stator voltage equations in the *d*-*q* synchronous rotating reference frame are given by [24]:

(1) $$\left\{\begin{array}{c} u_{d}=Ri_{d}+L_{d}\frac{di_{d}}{dt}-\omega_{e}\psi_{q}\\ u_{q}=Ri_{q}+L_{q}\frac{di_{q}}{dt}+\omega_{e}\psi_{d} \end{array}\right.$$

where $u_{d}$ and $u_{q}$ denote the stator voltage components along the *d*- and *q*-axes, respectively; $i_{d}$ and $i_{q}$ are the corresponding stator current components; $\psi_{d}$ and $\psi_{q}$ are the stator flux linkages; *R* is the stator resistance; and $\omega_{e}$ is the electrical-angular velocity.

The flux linkage equations in the *d*-*q* frame are given by [25]

(2) $$\left\{\begin{array}{c} \psi_{d}=L_{d}i_{d}+\psi_{f}\\ \psi_{q}=L_{q}i_{q} \end{array}\right.$$

where $L_{d}$ and $L_{q}$ are the *d*- and *q*-axis inductances, respectively, and $\psi_{f}$ is the rotor permanent magnet flux linkage along the *d*-axis.

The electromagnetic torque $T_{e}$ and the rotor mechanical equation are given by [26]

(3) $$T_{e}=\frac{3}{2}P_{n}\left(\psi_{f}i_{q}+\left(L_{d}-L_{q}\right)i_{d}i_{q}\right)$$

(4) $$\frac{J}{P_{n}}\frac{d\omega_{e}}{dt}=T_{e}-T_{L}-\frac{B}{P_{n}}\omega_{e}$$

where $T_{e}$ is the electromagnetic torque, $T_{L}$ is the load torque, $P_{n}$ is the number of pole pairs, *J* is the rotor inertia, and *B* is the viscous damping coefficient.

In practical surface-mounted PMSM drive systems, the $i_{d}^{*}=0$ control strategy is typically applied. As illustrated in Figure 1, the overall control structure integrates the sensorless speed estimation scheme to obtain high-precision rotor position and speed information, thereby satisfying the control requirements of the PMSM drive.

## 3. Conventional FCS-MPC Speed Estimation Method

In sensorless speed estimation approaches based on FCS-MPC, the electrical angle is typically treated as an optimization variable, with the optimal angle determined by searching a finite candidate set through a formulated cost function [27]. Considering the well-defined spatial relationship between the stator flux linkage and rotor position, the flux linkage data serve as the basis for constructing the electrical-angle estimation model. Conventional FCS-MPC speed estimation methods generally adopt a fixed-step discretized search strategy for the electrical angle. Specifically, a set of candidate angles is formed around the current estimate, and the best candidate is determined based on the cost function evaluation as the estimation outcome for the present sampling interval.

### 3.1. Stator Flux Linkage Calculation Model

To determine the electrical angle, a stator flux linkage formulation is initially established. Within the two-phase stationary αβ coordinate system, the stator flux linkage is obtained through the integration of the stator voltage equations. By disregarding inverter dead-time effects and high-frequency disturbances, the stator flux linkage can be represented as [28]

(5) $$\left\{\begin{array}{c} \psi_{\alpha }=\int_{0}^{t}\left(u_{\alpha }-Ri_{\alpha }\right)d\tau +\psi_{f}\cos\theta_{e0}\\ \psi_{\beta }=\int_{0}^{t}\left(u_{\beta }-Ri_{\beta }\right)d\tau +\psi_{f}\sin\theta_{e0} \end{array}\right.$$

where $\psi_{\alpha }$ and $\psi_{\beta }$ denote the stator flux linkage components along the α- and β-axes; $u_{\alpha }$, $u_{\beta }$ and $i_{\alpha }$, $i_{\beta }$ are the corresponding stator voltage and current components; and $\theta_{e0}$ is the initial electrical angle.

From Equation (5), the stator flux linkages expressed in stationary αβ coordinates are obtained. A transformation from the fixed αβ frame into the rotating dq system is performed to develop the rotor position estimation algorithm. Within this dq-coordinate system, the resulting flux components directly reflect the alignment of the rotor magnetic field direction with the stator flux. Therefore, the dq-axis flux quantities are calculated via the Park transformation as

(6) $$\left[\begin{array}{c} \psi_{d}\\ \psi_{q} \end{array}\right]=\left[\begin{array}{cc} \cos\theta_{e} & \sin\theta_{e}\\ -\sin\theta_{e} & \cos\theta_{e} \end{array}\right]\left[\begin{array}{c} \psi_{\alpha }\\ \psi_{\beta } \end{array}\right]$$

where $\psi_{d}$ and $\psi_{q}$ are the flux linkage components in the synchronous rotating frame, and $\theta_{e}$ is the electrical angle.

Using the flux linkage model and coordinate transformation relationships, a mapping between the stator flux and the rotor electrical position is developed, providing a theoretical foundation for constructing the candidate angle set and designing the cost function.

### 3.2. Construction of Flux Linkage Error Cost Function

In conventional FCS-MPC-based sensorless speed estimation methods, it is necessary to construct an evaluation index that reflects the electrical-angle estimation error, enabling selection of the optimal angle from the candidate set [29]. Considering that the stator flux linkage vector exhibits a well-defined spatial relationship with the rotor magnetic field, any error in the electrical-angle estimation distorts their relative alignment. Accordingly, the flux linkage information can be employed to construct an error function characterizing the estimation error of the electrical angle.

Let the true stator flux linkage in the synchronous rotating reference frame be denoted as

(7) $$\psi_{s}=\left[\begin{array}{c} \psi_{d}\\ \psi_{q} \end{array}\right]$$

and the corresponding estimated flux linkage is given by

(8) $${\hat{\psi }}_{s}=\left[\begin{array}{c} {\hat{\psi }}_{d}\\ {\hat{\psi }}_{q} \end{array}\right]$$

where ${\hat{\psi }}_{d}$ and ${\hat{\psi }}_{q}$ denote the estimated flux linkage components along the *d*- and *q*-axes, respectively.

When an estimation error Δθ occurs in the electrical angle, a spatial rotational relationship arises between the true and estimated flux linkage vectors. According to the coordinate transformation relationship, the estimated flux linkage can be expressed as the true flux linkage rotated by this angular deviation:

(9) $${\hat{\psi }}_{s}=\left[\begin{array}{cc} \cos\Delta \theta & \sin\Delta \theta \\ -\sin\Delta \theta & \cos\Delta \theta \end{array}\right]\psi_{s}$$

Expanding Equation (9) yields

(10) $$\left\{\begin{array}{c} {\hat{\psi }}_{d}=\psi_{d}\cos\Delta \theta +\psi_{q}\sin\Delta \theta \\ {\hat{\psi }}_{q}=-\psi_{d}\sin\Delta \theta +\psi_{q}\cos\Delta \theta \end{array}\right.$$

To construct an error signal reflecting the angular deviation, the cross-product relationship between the true and estimated flux linkage vectors is utilized. Based on the geometric relationship of these vectors, the flux linkage error is defined as [30]

(11) $$\varepsilon =\psi_{d}{\hat{\psi }}_{q}-{\hat{\psi }}_{d}\psi_{q}$$

Substituting Equation (10) into Equation (11) yields

(12) $$\varepsilon =-(\psi_{d}^{2}+\psi_{q}^{2})\sin\Delta \theta$$

Since the magnitude of the stator flux linkage satisfies $|\psi_{s}{|}^{2}=\psi_{d}^{2}+\psi_{q}^{2}$, the flux linkage error can therefore be expressed as

(13) $$\varepsilon =-|\psi_{s}{|}^{2}\sin\Delta \theta$$

It can be seen that the flux linkage error is proportional to sin(Δθ), i.e., ε∝sin(Δθ). For small angular deviations, sin(Δθ)≈Δθ, which leads to $\varepsilon \approx -|\psi_{s}{|}^{2}\Delta \theta$. From this relationship, the flux linkage error is approximately linearly related to the electrical-angle error. Therefore, the flux linkage error can serve as an evaluation index, and online estimation of the electrical angle can be performed by minimizing the flux linkage error.

On the basis of the above analysis, the squared flux error is adopted as the cost function:

(14) $$J={\left(\psi_{d}{\hat{\psi }}_{q}-{\hat{\psi }}_{d}\psi_{q}\right)}^{2}$$

The cost function is evaluated for each candidate angle within the candidate set, and the angle yielding the minimum cost is chosen as the optimal electrical-angle estimate for the current sampling period.

### 3.3. Construction of Candidate Electrical-Angle Set and Optimal Angle Search in Conventional FCS-MPC

After establishing the flux linkage model and constructing the cost function, it is necessary to search for the electrical angle that minimizes the cost function within a finite candidate set, thereby enabling online estimation of the electrical angle. Within the FCS-MPC framework, the optimization variable is typically selected via a discrete search over a candidate set. Consequently, the continuously varying electrical angle is discretized into a finite candidate set, from which the optimal estimate is determined.

Considering that the electrical-angle variation within a single sampling period is limited, conventional FCS-MPC speed estimation methods construct a local candidate angle set centered around the optimal electrical-angle estimate $\theta_{e,\mathrm{base}}$ obtained in the previous sampling period. To simplify the search process and facilitate real-time implementation, multiple candidate angles are symmetrically selected around $\theta_{e,\mathrm{base}}$ using a fixed step-size $\Delta \theta_{e}$, such that the interval between adjacent candidate angles remains constant. Therefore, the candidate electrical angle set for the current sampling period is expressed as follows:

(15) $$\theta_{e,i}=\theta_{e,\mathrm{base}}+\Delta \theta_{e}\left(i-4\right),\;i=0,1,\ldots ,8$$

where $\Delta \theta_{e}$ denotes the fixed angular search step, and $\theta_{e,i}$ represents the *i*-th candidate electrical angle.

**Figure 2:**
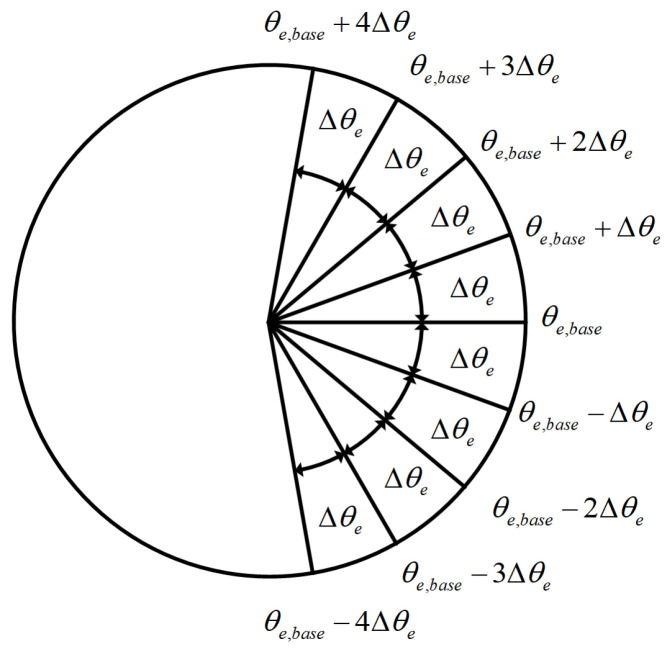
Schematic diagram of the conventional fixed-step candidate electrical-angle set construction.

In the above candidate set, nine candidate angles are constructed centered at $\theta_{e,\mathrm{base}}$, and the search interval is discretized using a fixed step size. Each candidate angle $\theta_{e,i}$ corresponds to a flux linkage estimate, from which the corresponding cost function value can be calculated. The construction of the fixed-step candidate angle set is illustrated in Figure 2.

Within this candidate set, nine angles are constructed, each corresponding to a flux linkage estimate. By substituting $\theta_{e,i}$ into Equation (6), the estimated flux linkage in the synchronous dq frame is obtained as follows:

(16) $$\left[\begin{array}{c} {\hat{\psi }}_{d,i}\\ {\hat{\psi }}_{q,i} \end{array}\right]=\left[\begin{array}{cc} \cos\theta_{e,i} & \sin\theta_{e,i}\\ -\sin\theta_{e,i} & \cos\theta_{e,i} \end{array}\right]\left[\begin{array}{c} \psi_{\alpha }\\ \psi_{\beta } \end{array}\right]$$

The cost function corresponding to each candidate angle is calculated according to Equation (14):

(17) $$J_{i}={\left(\psi_{d}{\hat{\psi }}_{q,i}-{\hat{\psi }}_{d,i}\psi_{q}\right)}^{2}$$

By comparing the cost function values for all candidate angles, the angle associated with the lowest cost is identified as the optimal electrical-angle estimate for the current sampling period:

(18) $$\theta_{e}=\arg\min J_{i}$$

where $J_{i}$ denotes the cost function value corresponding to the *i*-th candidate angle.

**Figure 3:**
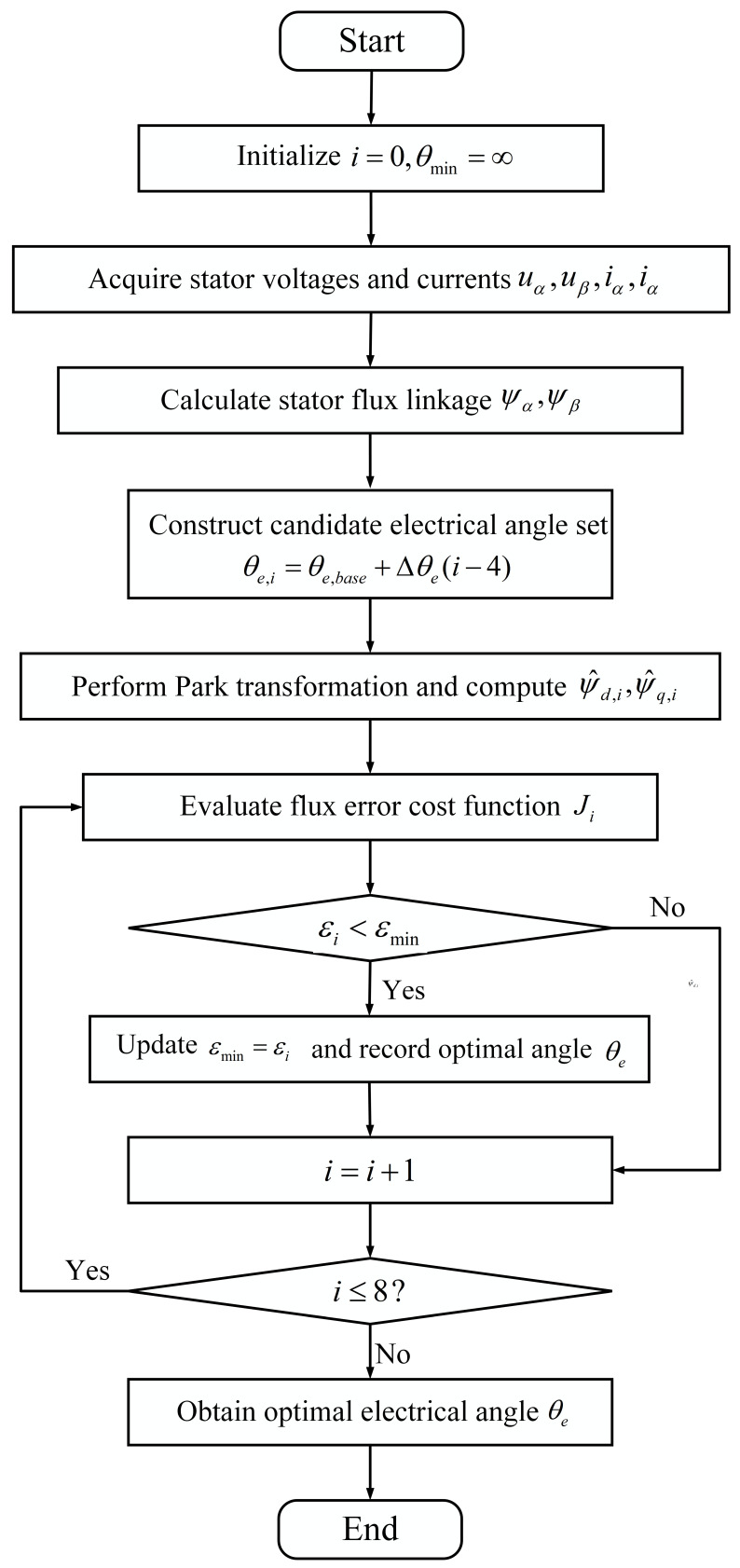
Flowchart of the fixed-step electrical-angle search algorithm FCS-MPC.

Using this fixed-step candidate set, the optimal electrical angle is determined online and can be used to update the base angle $\theta_{e,\mathrm{base}}$ for the next sampling period, as well as provide a basis for subsequent rotor speed estimation. Figure 2 illustrates the construction of the fixed-step candidate angle set, and Figure 3 presents the flowchart of the fixed-step electrical-angle search algorithm.

Although the fixed-step enumeration search method is straightforward to implement, it presents an inherent trade-off between search accuracy and computational complexity. A large step size reduces computational complexity but can deteriorate the estimation accuracy of the electrical angle, whereas a smaller step size improves accuracy at the expense of intensified resource consumption and degraded execution efficiency. Consequently, the conventional fixed-step search strategy cannot simultaneously achieve rapid transient performance and superior steady-state accuracy, limiting its applicability in high-performance sensorless control. To overcome these limitations, the following section introduces an electrical-angle optimization method based on an adaptive step-size combined with a ternary search strategy.

## 4. Design of Adaptive Step-Size and Ternary Search-Based FCS-MPC Speed Estimation

Conventional fixed-step FCS-MPC speed estimation methods are easy to implement, but the fixed search step size limits the balance between dynamic response, estimation accuracy, and computational complexity. To address this limitation, this section presents an adaptive step-size and ternary search-based FCS-MPC speed estimation approach. In the proposed scheme, the electrical-angle search increment is adaptively regulated according to the flux linkage error, while a ternary search algorithm is employed to improve the optimization performance of the electrical-angle estimation procedure and decrease computational complexity.

### 4.1. Analysis of the Fixed-Step Electrical-Angle Search Method

To introduce the adaptive step-size search strategy, the limitations of the conventional fixed-step electrical-angle search method are first analyzed. In the conventional method, a candidate angle set is constructed around the optimal electrical angle $\theta_{e,\mathrm{base}}$ obtained in the preceding sampling interval, and the optimal angle is determined via exhaustive evaluation of the cost function. Although this method is easy to implement, the fixed search step-size $\Delta \theta_{e}$ introduces a compromise between transient performance and steady-state estimation accuracy.

**Figure 4:**
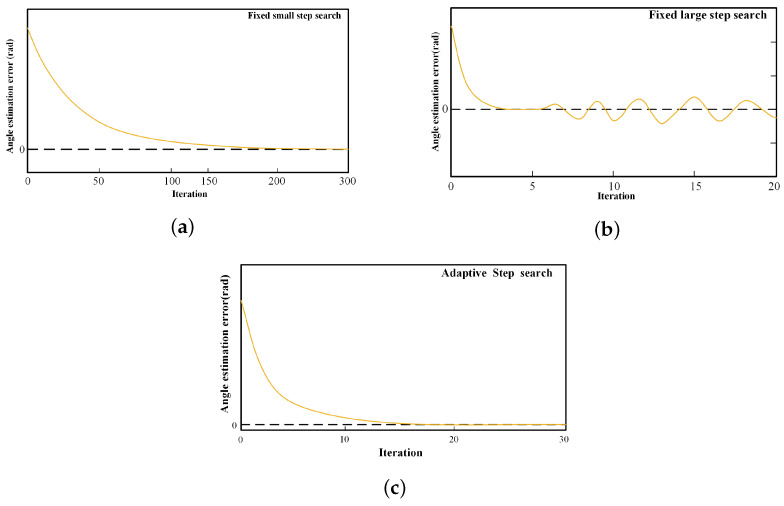
Schematic diagrams of the angle search process under different step-size strategies. (**a**) Fixed small-step search. (**b**) Fixed large-step search. (**c**) Adaptive step-size search.

A small step size improves steady-state estimation accuracy but limits the search range, which may lead to large estimation errors under transient conditions. Conversely, a large step size expands the search range and improves dynamic convergence, but increases discretization errors and angular oscillations. As illustrated in Figure 4, the adaptive step-size strategy employs a larger step during large estimation errors and gradually reduces the step near the optimum, thereby achieving both fast convergence and high steady-state accuracy.

From the above analysis, it is evident that the fixed-step search strategy cannot effectively balance search efficiency and angle estimation accuracy. Therefore, an enhanced electrical-angle search strategy capable of dynamically adjusting the search step size according to the system’s operating state is required. To address this, an adaptive step-size adjustment strategy is proposed in the subsequent section to enhance the performance of the sensorless speed estimation algorithm.

### 4.2. Adaptive Electrical-Angle Step-Size Adjustment Strategy

To overcome the trade-off between dynamic response and steady-state accuracy discussed in Section 4.1, the electrical-angle search step is adjusted adaptively according to the estimation error. In Section 3, the relation of the flux linkage error with the rotor angle error is established as follows [31]:

(19) $$\varepsilon =-{\left|\psi_{s}\right|}^{2}\sin\Delta \theta$$

For small angle deviations, the error can be approximated as follows:

(20) $$\varepsilon \approx -{\left|\psi_{s}\right|}^{2}\Delta \theta$$

Thus, the flux linkage error is approximately proportional to the electrical-angle error. After eliminating the influence of the stator flux magnitude, the normalized error is defined as follows:

(21) $$\varepsilon_{\mathrm{norm}}=\frac{\left|\varepsilon_{\min}\right|}{\varepsilon_{0}+{\left|\psi_{s}\right|}^{2}}$$

where $\varepsilon_{\min}$ denotes the minimum flux linkage error within the candidate angle set, $\varepsilon_{0}$ is a minor constant introduced for avoiding division-by-zero issues, and $|\psi_{s}|$ is the magnitude of the stator flux linkage.

The adaptive electrical-angle search step size is then defined as follows:

(22) $$\Delta \theta_{\mathrm{auto}}=\Delta \theta_{\min}+k\varepsilon_{\mathrm{norm}}$$

where $\Delta \theta_{\min}$ is the minimum search step size and *k* is a coefficient controlling the step-size adjustment range. Substituting Equation (21) into Equation (22) yields the following explicit expression:

(23) $$\Delta \theta_{\mathrm{auto}}=\Delta \theta_{\min}+k\frac{|\varepsilon_{\min}|}{\varepsilon_{0}+{\left|\psi_{s}\right|}^{2}}$$

Accordingly, a larger normalized flux linkage error results in a larger search step, which expands the candidate angle range and accelerates convergence. As the estimation error decreases, the search step is automatically reduced, leading to a denser candidate angle distribution and improved steady-state accuracy.

In this study, the minimum search step size was set to $\Delta \theta_{\min}=1{}^{\circ}$. The adaptive coefficient *k* was selected by considering both the local unimodality requirement of the ternary search and the maximum electrical-angle variation within one sampling period. Since the adaptive step size is defined by Equation (22), the coefficient *k* determines the upper adjustment range of the electrical-angle search step.

To satisfy the local-unimodality requirement of the ternary search, the local search interval should be restricted within the locally unimodal region, whose detailed justification is provided in Section 4.3. In the proposed method, the local search interval is centered at $\theta_{e,\mathrm{base}}$ and extends by two adaptive step sizes on each side, where the coefficient 2 is a predefined search-margin factor; therefore, its half-width is $2\Delta \theta_{\mathrm{auto}}$.

For the local-unimodality constraint, only the maximum half-width of the search interval is considered. Therefore, the following condition should be satisfied:

(24) $$2\Delta \theta_{\mathrm{auto},\max}<90{}^{\circ}$$

where $\Delta \theta_{\mathrm{auto},\max}$ denotes the maximum value of the adaptive electrical-angle search step.

Under large-error conditions, the normalized flux-linkage error approaches unity, and the adaptive search step approaches its upper range. Considering $\Delta \theta_{\mathrm{auto},\max}\approx \Delta \theta_{\min}+k$, the upper bound of *k* can be obtained as follows:

(25) $$\begin{array}{cc} 2\left(\Delta \theta_{\min}+k\right) & <90{}^{\circ}\\ k & <\frac{90{}^{\circ}}{2}-\Delta \theta_{\min}\\ & =\frac{90{}^{\circ}}{2}-1{}^{\circ}\\ & =44{}^{\circ} \end{array}$$

Therefore, when $\Delta \theta_{\min}=1{}^{\circ}$, the adaptive coefficient should be selected below 44° to preserve the local unimodality required by the ternary search.

In addition to the local-unimodality constraint, the maximum electrical-angle variation within one sampling period should also be considered to ensure that the local search interval is sufficiently wide to cover the angle variation under high-speed or transient conditions. According to the motor parameters listed in Table 1, the maximum mechanical speed is $\omega_{\max}=3000\;r/\min$, the number of pole pairs is $P_{n}=4$, and the sampling period is $T_{s}=100\;\mu s$. Therefore, the maximum electrical-angle increment within one sampling period can be calculated as follows:

(26) $$\begin{array}{cc} \Delta \theta_{e,\max} & =P_{n}\frac{2\pi \omega_{\max}}{60}T_{s}\\ & =4\times \frac{2\pi \times 3000}{60}\times 10^{-4}\\ & =0.1257\;\mathrm{rad}\\ & \approx 7.2{}^{\circ} \end{array}$$

where $\Delta \theta_{e,\max}$ denotes the maximum possible electrical-angle variation within one sampling period.

To ensure that the local search interval can cover this maximum electrical-angle variation, its half-width should satisfy

(27) $$2\left(\Delta \theta_{\min}+k\varepsilon_{\mathrm{norm}}\right)\geq \Delta \theta_{e,\max}$$

Under large-error conditions, $\varepsilon_{\mathrm{norm}}$ can approach unity. Therefore, the lower requirement for *k* can be obtained as follows:

(28) $$\begin{array}{cc} 2\left(\Delta \theta_{\min}+k\right) & \geq \Delta \theta_{e,\max}\\ k & \geq \frac{\Delta \theta_{e,\max}}{2}-\Delta \theta_{\min}\\ & =\frac{7.2{}^{\circ}}{2}-1{}^{\circ}\\ & =2.6{}^{\circ} \end{array}$$

Consequently, by combining the local-unimodality constraint and the search-coverage requirement, the feasible range of *k* can be approximately determined as follows:

(29) 2.6°≤k<44°

Based on this range, k=10° was adopted in this study. Under large-error conditions, the corresponding maximum adaptive search step is approximately

(30) $$\Delta \theta_{\mathrm{auto},\max}\approx \Delta \theta_{\min}+k=1{}^{\circ}+10{}^{\circ}=11{}^{\circ}$$

Accordingly, the maximum half-width of the local search interval is

(31) $$2\Delta \theta_{\mathrm{auto},\max}=2\times 11{}^{\circ}=22{}^{\circ}$$

This value is substantially smaller than the 90° boundary of the locally unimodal region and is also sufficiently larger than the maximum electrical-angle increment of 7.2° within one sampling period. Therefore, k=10° ensures the validity of the ternary search while providing sufficient coverage for high-speed electrical-angle variations. Meanwhile, it avoids an excessively wide search interval, thereby achieving a practical compromise between transient convergence capability and steady-state angular stability.

The small constant $\varepsilon_{0}$ was introduced to avoid division-by-zero and numerical ill-conditioning when the stator flux magnitude is very small. According to the motor parameters in Table 1, the permanent-magnet flux linkage is $\psi_{f}=0.00838\;\mathrm{Wb}$, and the corresponding nominal value of $|\psi_{s}{|}^{2}$ is approximately $7.02\times 10^{-5}\;{\mathrm{Wb}}^{2}$. Therefore, $\varepsilon_{0}=1\times 10^{-8}\;{\mathrm{Wb}}^{2}$ was adopted, which is much smaller than the nominal squared flux-linkage value and has little influence on the normalized error under normal operating conditions.

### 4.3. Ternary Search-Based Electrical-Angle Optimization Method

In the conventional electrical-angle estimation method described in Section 3, the cost function is evaluated over a predefined candidate angle set, and the angle associated with the lowest cost is selected as the best estimate. Although this exhaustive search is straightforward, its computational complexity increases with the number of candidate angles, which may limit real-time performance when a finer search resolution is required.

To reduce the computational complexity, a ternary search method is introduced to optimize the electrical angle within a local search interval. Since the cost function $J(\theta_{e})$ exhibits a locally unimodal characteristic around the true electrical angle, the ternary search can be applied to progressively narrow the search interval. In this study, the search is performed locally around the optimal angle obtained in the previous sampling period, which is reasonable due to the continuity of the electrical angle between consecutive sampling instants.

The applicability of the ternary search is further justified by analyzing the local unimodality of the cost function. According to the flux-linkage error relation derived in Section 3, the cost function can be expressed as follows:

(32) $$J\left(\theta_{e,i}\right)=\varepsilon^{2}={\left|\psi_{s}\right|}^{4}\sin^{2}\left(\theta_{e,i}-\theta_{e,\mathrm{act}}\right)$$

where $\theta_{e,i}$ denotes the *i*th candidate electrical angle evaluated during the ternary search process, and $\theta_{e,\mathrm{act}}$ denotes the actual electrical angle at the current sampling instant.

It should be noted that this function is not globally unimodal over the whole electrical-angle range because of the periodicity of the sine function. However, within a local interval around $\theta_{e,\mathrm{act}}$, the function has only one minimum. When the angle error satisfies $-\pi /2<\theta_{e,i}-\theta_{e,\mathrm{act}}<0$, $J(\theta_{e,i})$ decreases as $\theta_{e,i}$ approaches $\theta_{e,\mathrm{act}}$; when $0<\theta_{e,i}-\theta_{e,\mathrm{act}}<\pi /2$, $J(\theta_{e,i})$ increases as $\theta_{e,i}$ moves away from $\theta_{e,\mathrm{act}}$. Therefore, the cost function is locally unimodal around the actual electrical angle. With the adopted parameters, $\Delta \theta_{\mathrm{auto}}$ is normally bounded within a small range, and the local search interval $\left[\theta_{e,\mathrm{base}}-2\Delta \theta_{\mathrm{auto}},\theta_{e,\mathrm{base}}+2\Delta \theta_{\mathrm{auto}}\right]$ remains much narrower than π. Therefore, when the previous electrical-angle estimate is close to the actual angle, the search interval lies within the locally unimodal region.

In the proposed method, the ternary search is therefore performed only within this local search interval rather than over the entire electrical-angle range. With the selected adaptive step-size parameters, the local unimodality condition required by ternary search can be satisfied under normal operating conditions.

The influence of different speeds and load torques can be interpreted from the cost-function expression. Rotor speed mainly affects the variation rate of the electrical angle, while load torque changes the stator current and the magnitude of the stator flux linkage. These factors may change the amplitude term $|\psi_{s}{|}^{4}$, but they do not change the basic $\sin^{2}\left(\theta_{e,i}-\theta_{e,\mathrm{act}}\right)$ structure of the cost function. Therefore, the local single-valley characteristic around the actual electrical angle can still be maintained under different speed and load conditions.

When measurement noise and parameter variations are considered, the practical cost function can be regarded as a perturbed form of the ideal cost function:

(33) $$J\left(\theta_{e,i}\right)=J_{0}\left(\theta_{e,i}\right)+\Delta J\left(\theta_{e,i}\right)$$

where $\Delta J(\theta_{e,i})$ represents the perturbation caused by current and voltage measurement noise, stator resistance variation, inductance mismatch, inverter nonlinearity, and other non-ideal factors. These factors may slightly distort the cost-function curve or shift the minimum point. However, as long as the perturbation is bounded and does not introduce additional local extrema within the adopted local search interval, the cost function can still be regarded as locally unimodal. Therefore, under normal operating conditions and bounded perturbations, the cost function can still be regarded as locally unimodal within the adopted local search interval.

Based on the adaptive step-size $\Delta \theta_{\mathrm{auto}}$ obtained in Section 4.2, the search interval for the current sampling period is defined as follows:

(34) $$\left[\theta_{e,L},\theta_{e,U}\right]=\left[\theta_{e,\mathrm{base}}-2\Delta \theta_{\mathrm{auto}},\;\theta_{e,\mathrm{base}}+2\Delta \theta_{\mathrm{auto}}\right]$$

where $\theta_{e,L}$ and $\theta_{e,U}$ denote the minimum and maximum limits of the optimization range, respectively.

Within this interval, two partition points are defined as follows:

(35) $$\begin{array}{cc} \theta_{1} & =\theta_{e,L}+\frac{\theta_{e,U}-\theta_{e,L}}{3}\\ \theta_{2} & =\theta_{e,U}-\frac{\theta_{e,U}-\theta_{e,L}}{3} \end{array}$$

The cost values $J(\theta_{1})$ and $J(\theta_{2})$ are then computed by substituting $\theta_{1}$ and $\theta_{2}$ into the cost function. For minimization, if $J\left(\theta_{1}\right)<J\left(\theta_{2}\right)$, the upper bound is updated as follows:

(36) $$\theta_{e,U}=\theta_{2}$$

Otherwise, the optimal angle lies in the right subinterval, and the interval is updated as follows:

(37) $$\theta_{e,L}=\theta_{1}$$

This process is repeated until the interval width satisfies

(38) $$\theta_{e,U}-\theta_{e,L}<\Delta \theta_{\min}$$

Finally, the optimal electrical-angle estimate is obtained as follows:

(39) $$\theta_{e}^{*}=\frac{\theta_{e,L}+\theta_{e,U}}{2}$$

To further quantify the relationship between the ternary search iteration number, search accuracy, and computational complexity, the interval reduction process is analyzed as follows. Let the initial search interval width be $W_{0}$. According to Equation (34), $W_{0}$ can be expressed as follows:

(40) $$W_{0}=\theta_{e,U}-\theta_{e,L}=4\Delta \theta_{\mathrm{auto}}$$

In each ternary search iteration, one third of the current interval is discarded. Therefore, after *m* iterations, the remaining search interval width becomes

(41) $$W_{m}={\left(\frac{2}{3}\right)}^{m}W_{0}=4\Delta \theta_{\mathrm{auto}}{\left(\frac{2}{3}\right)}^{m}$$

Since the final electrical-angle estimate is obtained as the midpoint of the remaining interval, the maximum angular uncertainty caused by the finite iteration number can be bounded by

(42) $$E_{m}\leq \frac{W_{m}}{2}=2\Delta \theta_{\mathrm{auto}}{\left(\frac{2}{3}\right)}^{m}$$

Thus, the search accuracy improves exponentially with the iteration number. Meanwhile, two cost-function evaluations are required in each iteration. Therefore, the number of cost-function evaluations for *m* ternary search iterations is

(43) $$C_{m}=2m$$

If the termination condition $W_{m}<\Delta \theta_{\min}$ is required, the minimum iteration number can be estimated as follows:

(44) $$m\geq \left\lceil \frac{\ln\left(4\Delta \theta_{\mathrm{auto}}/\Delta \theta_{\min}\right)}{\ln(3/2)}\right\rceil$$

The above analysis indicates that increasing *m* reduces the remaining search interval and improves the search accuracy, but it also increases the computational burden. Therefore, in practical real-time implementation, a fixed maximum iteration number is adopted to ensure deterministic computational cost within each sampling period, and the maximum iteration number is set to 4 in this study. When m=4, the remaining interval is reduced to ${\left(2/3\right)}^{4}=16/81$, specifically about 19.75% of the initial interval, while only 2m=8 cost-function evaluations are required. This is lower than the nine evaluations required by the conventional 9-point enumeration method. Therefore, under the adopted implementation, four iterations provide a suitable trade-off between search accuracy and computational complexity, which is also consistent with the comparative results in Table 2.

**Figure 5:**
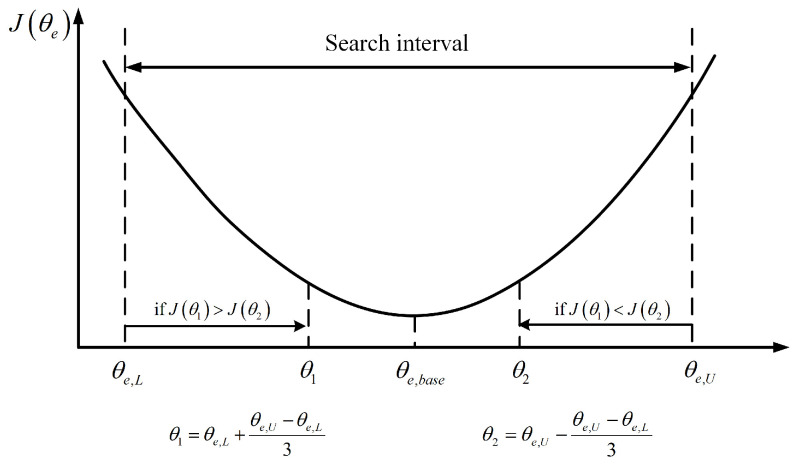
Schematic illustration of the ternary search process.

**Figure 6:**
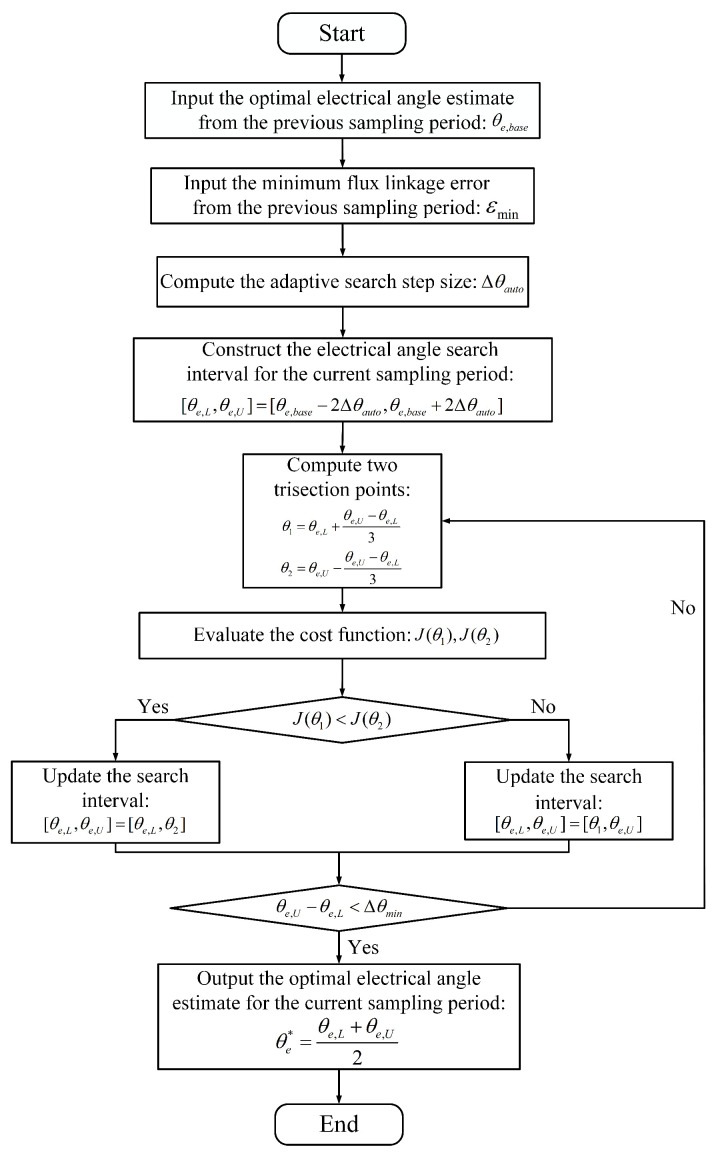
Flowchart of the optimal electrical-angle estimation based on ternary search.

Figure 5 illustrates the interval refinement process of the ternary search, while Figure 6 presents the corresponding flowchart of the ternary search-based electrical-angle optimization method.

### 4.4. Rotor Speed Estimation Method

After obtaining the optimal electrical-angle estimate, the rotor electrical speed is calculated from the angle increment between two consecutive sampling instants. Let $\theta_{e}\left(k\right)$ denote the electrical-angle estimate at the *k*-th sampling instant. The increment of the electrical-angle is given by

(45) $$\Delta \theta_{e}\left(k\right)=\theta_{e}\left(k\right)-\theta_{e}\left(k-1\right)$$

Direct speed estimation based on consecutive angle differences is sensitive to sampling noise and angle jitter. Therefore, a moving average filter is applied to the angle increment as follows:

(46) $$\Delta \theta_{\mathrm{avg}}\left(k\right)=\frac{1}{N}\sum_{i=0}^{N-1}\Delta \theta_{e}\left(k-i\right)$$

where *N* is the length of the moving average window.

In this paper, the window length of the moving average filter was set to N=200. The moving average filter is used to suppress high-frequency fluctuations caused by angle differencing. A smaller *N* leads to a faster response but weaker smoothing, whereas a larger *N* improves noise suppression but increases the estimation delay. Based on simulation and experimental tuning, N=200 was adopted to balance steady-state smoothness and dynamic response.

The rotor electrical-angular speed is then estimated by

(47) $${\hat{\omega }}_{e}\left(k\right)=\frac{\Delta \theta_{\mathrm{avg}}\left(k\right)}{T_{s}}$$

where $T_{s}$ denotes the system sampling period.

To further clarify the influence of the main parameters, the effects of *k*, $\varepsilon_{0}$, and *N* on the proposed method are summarized as follows. The coefficient *k* mainly affects the adaptive search range. A smaller *k* results in a conservative step-size adjustment and slower transient convergence, whereas a larger *k* accelerates the response but may increase steady-state fluctuation. The parameter $\varepsilon_{0}$ mainly affects numerical stability. When $\varepsilon_{0}$ is much smaller than $|\psi_{s}{|}^{2}$, its influence on the estimation performance is limited; however, an excessively large $\varepsilon_{0}$ reduces the magnitude of the normalized error and reduces the sensitivity of the adaptive step-size adjustment. The window length *N* determines the trade-off between speed smoothing and dynamic response. A smaller *N* provides a faster response but allows more high-frequency fluctuations to remain, whereas a larger *N* improves smoothing but introduces additional delay.

## 5. Simulation and Experimental Validation

To assess the proposed adaptive step-size and ternary search-based FCS-MPC speed estimation method, simulations and experiments were conducted. MATLAB/Simulink simulations were conducted to benchmark the proposed approach against the conventional fixed-step FCS-MPC scheme under various operating conditions. In addition, experimental validation was implemented using a PMSM test bench to verify the practical applicability of the proposed strategy.

### 5.1. Simulation Analysis

The conventional fixed-step FCS-MPC speed estimation method and the proposed adaptive step-size and ternary search-based method were implemented in MATLAB/Simulink for a comparison. The principal simulation configurations are presented in Table 1.

#### 5.1.1. Constant-Speed Performance and Iteration Analysis

The constant-speed performance was evaluated at a reference speed of 1000 r/min under no-load conditions, with the motor starting from standstill. In the proposed method, four ternary search iterations were used for local electrical-angle optimization.

**Figure 7:**
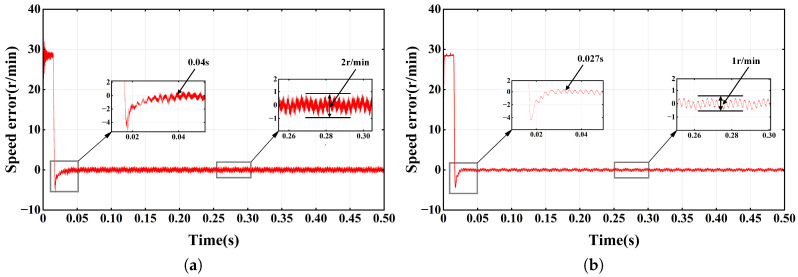
Comparison of speed estimation errors between fixed step-size and adaptive step-size FCS-MPC. (**a**) Fixed step-size. (**b**) Adaptive step-size.

**Figure 8:**
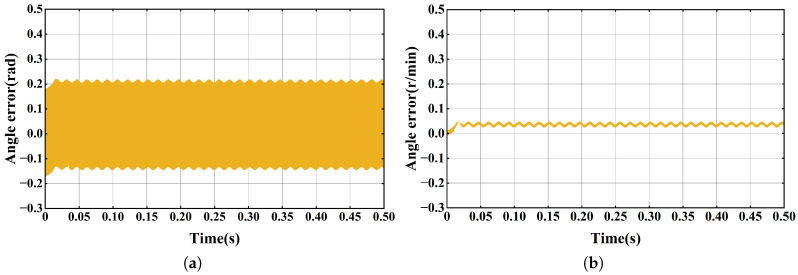
Comparison of position estimation errors between fixed step-size and adaptive step-size FCS-MPC. (**a**) Fixed step-size. (**b**) Adaptive step-size.

Figure 7a,b present the speed estimation errors of the fixed-step method and the proposed adaptive step-size method, respectively, while Figure 8a,b show the corresponding rotor position errors. At start-up, the peak speed error was reduced from approximately 30 r/min to 28 r/min by the proposed method. The convergence time decreases from 0.04 s to 0.027 s, corresponding to a 32.5% reduction. In steady state, the speed fluctuation is reduced from about 2 r/min to 1 r/min, indicating improved estimation stability.

The position error in Figure 8 shows a similar trend. Compared with the fixed-step method, the proposed method suppresses high-frequency oscillations and provides a smoother position estimation trajectory. These results indicate that the proposed method improves both speed and position estimation accuracy under constant-speed conditions.

**Figure 9:**
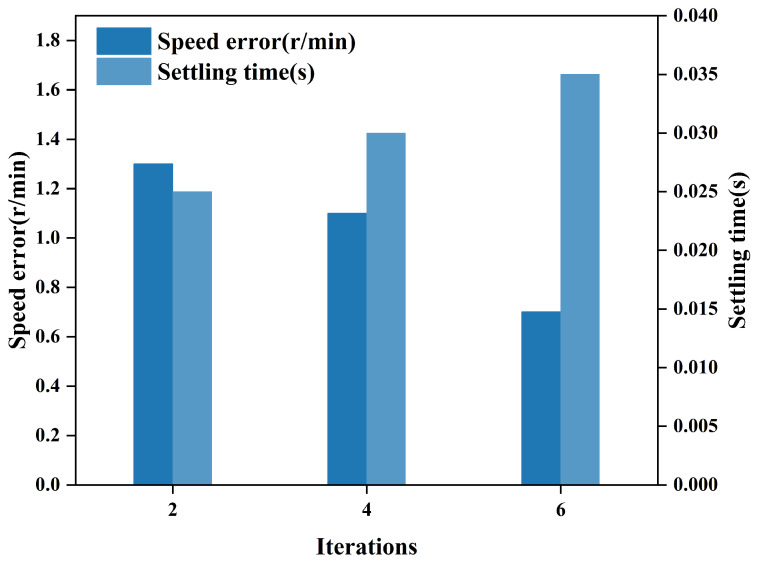
Effect of ternary search iteration number on speed error and settling time.

Figure 9 and Table 2 show the influence of ternary search iterations on estimation accuracy and settling time. Increasing the iteration number improves steady-state accuracy but also increases the number of cost-function evaluations. When the iteration number increases from 2 to 4, the speed error decreases from 1.3 r/min to 1.0 r/min with only a slight increase in settling time. Although 6 iterations further reduce the error to 0.7 r/min, they also increase the computational cost and settling time. Therefore, four iterations are selected as a compromise between accuracy, dynamic response, and real-time implementation.

Based on these results, four ternary search iterations are used in the following simulations and experiments.

#### 5.1.2. Simulation Under Load-Disturbance Condition

**Figure 10:**
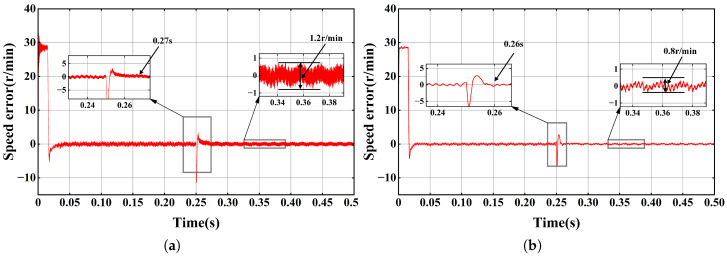
Comparison of speed estimation errors under sudden load condition. (**a**) Fixed step-size. (**b**) Adaptive step-size.

To evaluate disturbance rejection capability, an abrupt load torque of 0.1 N·m was imposed at t=0.25 s while the reference speed remained at 1000 r/min. As shown in Figure 10, maximum speed error decreases from approximately −12 r/min in the fixed-step method to about −6 r/min in the proposed method. The proposed method also recovers faster, with the error converging at approximately t=0.26 s.

In steady state, the speed fluctuation is reduced from about 1.2 r/min to 0.8 r/min. Since the position error exhibits a trend consistent with the speed error, only the speed estimation results are presented in the following load-disturbance and variable-speed analyses.

#### 5.1.3. Simulation Under Constant Load and Variable Speed Conditions

To further evaluate the transient tracking capability of the proposed method, a variable-speed simulation was performed under a constant load torque condition. The load torque was maintained at 0.1 N·m throughout the simulation. The reference speed was initially set to 1000 r/min, increased to 1500 r/min at t=0.25 s, and subsequently reduced to 800 r/min at t=0.5 s.

**Figure 11:**
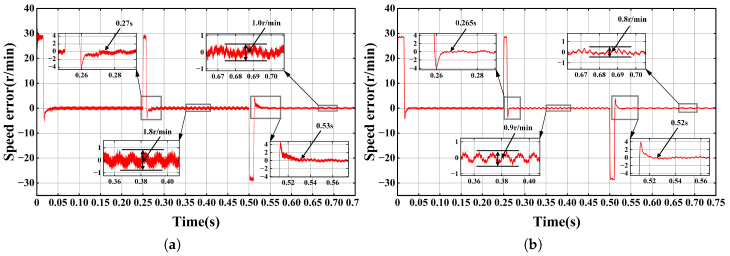
Comparison of speed estimation errors under constant-load variable-speed condition. (**a**) Fixed step-size. (**b**) Adaptive step-size.

As shown in Figure 11, speed step changes at t=0.25 s and t=0.5 s cause transient error fluctuations in both methods. The fixed-step method recovers at approximately t=0.27 s during acceleration and t=0.53 s during deceleration. In comparison, the proposed method exhibits smaller transient deviations and faster error convergence at both speed transitions.

In steady state, the speed error fluctuation is reduced from about 1.8 r/min in the fixed-step method to within 0.9 r/min in the proposed method, corresponding to an approximately 50% reduction. The proposed method also produces a smoother error trajectory with fewer oscillations, confirming its improved transient and steady-state estimation performance under constant-load variable-speed conditions.

### 5.2. Experimental Analysis

**Figure 12:**
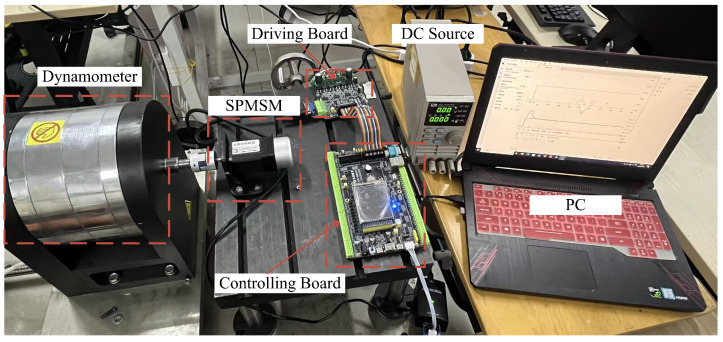
Experimental platform of the surface-mounted permanent magnet synchronous motor drive system.

**Figure 13:**
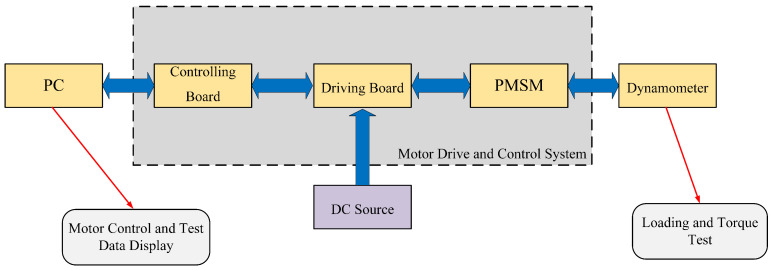
Overall control system structure.

To examine the practical feasibility of the proposed adaptive step-size and ternary search-based FCS-MPC speed estimation method, experiments were conducted on a surface-mounted PMSM platform. The experimental setup is illustrated in Figure 12, and the corresponding control framework is presented in Figure 13.

The sensorless vector control system was implemented on an STM32F407 microcontroller, in which the adaptive step-size FCS-MPC speed estimation algorithm was embedded for real-time execution. The PMSM model used in the controller was based on the standard *d*–*q* axis representation, where magnetic saturation, iron loss, and temperature-dependent parameter variation were neglected.

During the experiments, the rotor speed, current, and speed estimation error were recorded using a host computer. A constant load torque was applied by a dynamometer after the motor reached steady-state operation. Since the dynamometer applies load in discrete increments, abrupt load-disturbance tests are not conducted experimentally. The same PMSM parameters as those used in the simulation were adopted to ensure consistency between simulation and experimental validation.

#### 5.2.1. Effect of Fixed Step-Size Parameters on Speed Estimation Performance

**Figure 14:**
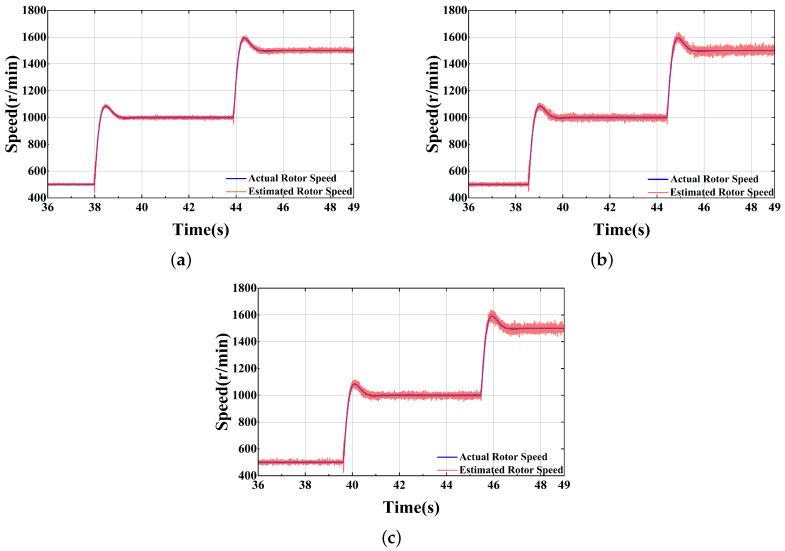
Speed response curves under different fixed step sizes. (**a**) 1°. (**b**) 10°. (**c**) 20°.

To investigate the influence of fixed step size on estimation performance, comparative experiments were conducted using the conventional fixed-step FCS-MPC method. The encoder-measured rotor speed was used as the reference, and fixed electrical-angle step sizes of 1°, 10°, and 20° were tested under stepwise speed transitions from 500 r/min to 1000 r/min and then to 1500 r/min. As shown in Figure 14, the estimated speed follows the reference speed under all three step-size settings.

**Figure 15:**
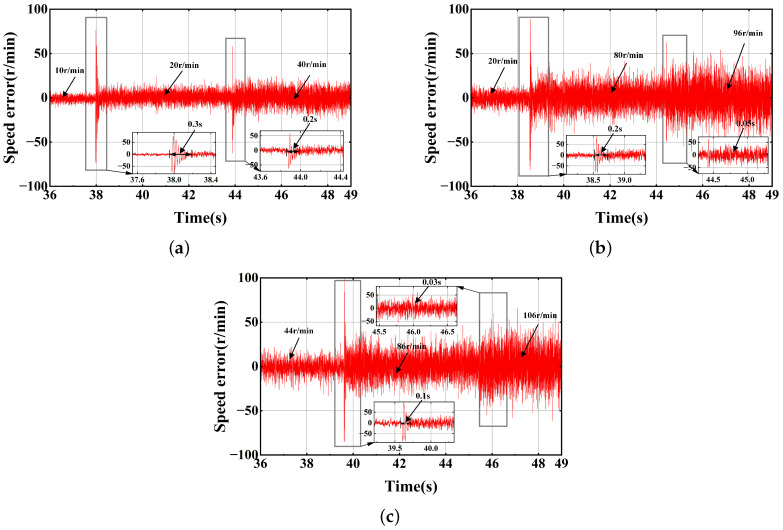
Speed estimation errors with different fixed electrical-angle step sizes. (**a**) 1°. (**b**) 10°. (**c**) 20°.

For a dynamic response, the 1° step size provides smooth convergence with small overshoot, although the settling duration is comparatively longer. As shown in the magnified region of Figure 15, the transitions from 500 r/min to 1000 r/min and from 1000 r/min to 1500 r/min require approximately 0.3 s and 0.2 s, respectively. Increasing the step size to 10° and 20° accelerates tracking, but also introduces larger transient fluctuations and oscillations. This indicates a trade-off between response speed and smoothness for fixed-step methods.

The steady-state speed errors under different step sizes are shown in Figure 15. At 1°, the steady-state deviations at 500 r/min, 1000 r/min, and 1500 r/min are approximately 10, 20, and 40 r/min, respectively. As the step size increases to 10° and 20°, the error range becomes larger, especially at medium and high speeds. These results confirm that a fixed step size cannot simultaneously achieve fast dynamic response and high steady-state accuracy.

#### 5.2.2. Experimental Validation of the Proposed Adaptive Step-Size and Ternary Search-Based Method

**Figure 16:**
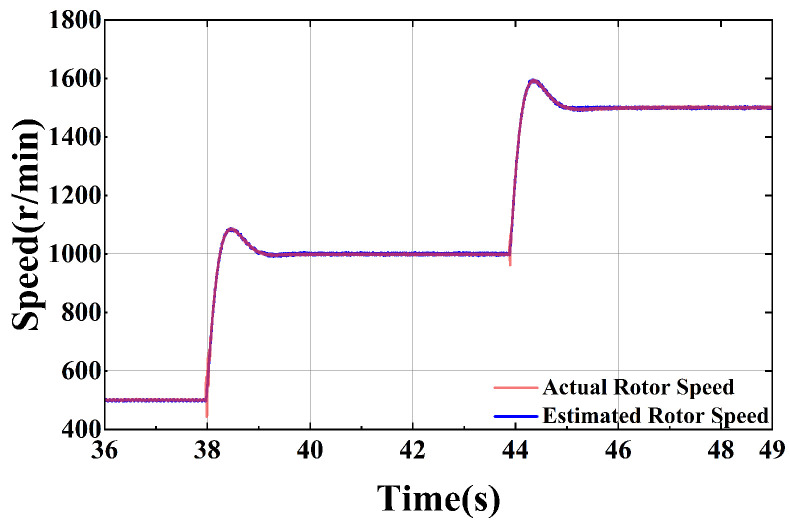
Speed response of the adaptive step-size method.

**Figure 17:**
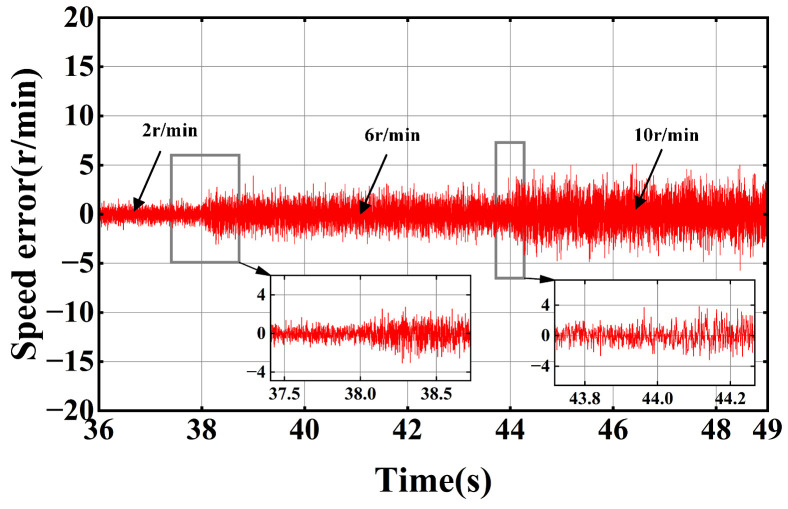
Speed estimation error of the adaptive step-size method.

Based on the above analysis, the proposed adaptive step-size and ternary search-based FCS-MPC method was further tested under the same speed transition conditions. Figure 16 and Figure 17 show the speed response and the corresponding estimation error, respectively. The estimated speed closely follows the encoder-measured speed during the transitions from 500 r/min to 1000 r/min and then to 1500 r/min, with small overshoot and fast convergence.

Compared with the fixed-step results in Figure 15, the proposed method achieves an improved trade-off between transient behavior and steady-state accuracy. During speed transitions, the proposed method maintains fast convergence, while in steady state, the estimation error is effectively constrained. These results experimentally verify the validity of the proposed adaptive step-size strategy.

To quantitatively evaluate the experimental speed estimation performance, the conventional fixed-step FCS-MPC methods with different step sizes and the proposed adaptive step-size and ternary search-based method are compared in Table 3. The comparison indices include the settling time during speed transitions, maximum speed estimation error, and steady-state speed estimation error. For the proposed method, the transient speed fluctuation is very small and the settling process is not clearly distinguishable from the experimental curve; therefore, its settling time is marked as “Negligible” in the table.

As shown in Table 3, the fixed-step methods show different trade-offs between dynamic response and steady-state accuracy. For the 500 r/min to 1000 r/min transition, increasing the fixed step size shortens the settling time from 0.3 s to 0.1 s, but increases the maximum speed error and steady-state speed error. A similar trend can also be observed during the 1000 r/min to 1500 r/min transition. In contrast, the proposed method exhibits negligible settling behavior because its transient speed fluctuation is very small, while the maximum and steady-state speed errors remain much lower than those of the fixed-step methods. These results further verify that the proposed adaptive step-size and ternary search-based method can improve the overall balance between transient response and steady-state estimation accuracy.

It should be noted that sudden load application and load rejection experiments were not conducted due to the loading mode limitation of the available dynamometer, which can only increase the load torque in small discrete increments and cannot generate an abrupt torque disturbance comparable to the simulation condition. Nevertheless, the expected load-disturbance performance of the proposed method can be partly supported by the simulation results in Section 5.1.2. When a sudden load disturbance occurs, the increased flux-linkage error enables the adaptive step-size strategy to enlarge the electrical-angle search range and accelerate error convergence. After the disturbance is suppressed, the search step is reduced to improve steady-state accuracy. Therefore, under the simulated load-disturbance condition, the proposed method is expected to provide improved disturbance rejection capability compared with the conventional fixed-step method. Further experimental validation using a loading device capable of fast torque step application and load rejection will be conducted in future work.

#### 5.2.3. Real-Time Computational Performance Analysis

To evaluate the real-time implementation feasibility of the proposed method, the computational performance was measured on the STM32F407 microcontroller used in the experimental platform. The execution time was measured within one sampling period, and the measured code segment includes the electrical-angle search, cost-function evaluation, adaptive step-size calculation, and speed estimation process. The CPU load was calculated based on the measured maximum execution time within one sampling period.

As shown in Table 4, the average execution time of the conventional fixed-step FCS-MPC method is 56μs, while that of the proposed adaptive step-size and ternary search-based method is reduced to 46μs, corresponding to a reduction of approximately 17.86%. The maximum execution time is also reduced from 60μs to 57μs. Under the adopted sampling period of $T_{s}=100\;\mu s$, the corresponding CPU load calculated from the maximum execution time decreases from 60% to 57%, indicating that both methods satisfy the real-time execution requirement.

Although the proposed method introduces additional adaptive step-size calculation and ternary search logic, its Flash usage increases only from 16,720 bytes to 16,748 bytes, and its RAM usage increases only from 2592 bytes to 2600 bytes. The additional memory consumption is therefore very small. These results demonstrate that the proposed method reduces the average computational time while maintaining low memory overhead, verifying its feasibility for real-time implementation on the STM32F407-based control platform.

## 6. Conclusions

This paper develops a sensorless speed estimation strategy for PMSM drives based on adaptive step-size regulation and ternary search-assisted FCS-MPC. By integrating adaptive electrical-angle step-size adjustment with ternary search optimization, the proposed strategy enhances the transient response and steady-state behavior of conventional fixed step-size FCS-MPC, while also reducing the computational burden. The feasibility and performance of the developed scheme are assessed through simulation and experimental tests under different operating conditions.

The experimental results show that the proposed method achieves fast speed convergence and improved estimation accuracy during speed transitions. For the speed transitions from 500r/min to 1000r/min and from 1000r/min to 1500r/min, the transient speed fluctuation of the proposed method is very small, and the corresponding settling time is negligible in the experimental curves. The maximum speed estimation errors are limited to 8r/min and 12r/min, and the steady-state speed estimation errors are 6r/min and 10r/min, respectively. Compared with the conventional fixed-step methods, the proposed method provides a better compromise between dynamic response and steady-state estimation accuracy. Moreover, the real-time computational performance is verified on the STM32F407 platform. The average execution time is reduced from 56μs to 46μs, corresponding to a reduction of approximately 17.86%, while the maximum execution time is reduced from 60μs to 57μs. Under the sampling period of $T_{s}=100\;\mu s$, the proposed method satisfies the real-time execution requirement with only a slight increase in Flash and RAM usage.

Future work will further investigate the robustness of the proposed method under practical non-ideal conditions, including motor-parameter variations such as stator-resistance and inductance variations, temperature-related parameter changes, inverter dead-time effects, current/voltage measurement noise, and load torque uncertainty. In addition, the applicability and limitations of the proposed flux-linkage-based sensorless speed estimation strategy under very-low-speed and near-zero-speed operating conditions will be further examined, since reliable sensorless estimation in these regions remains challenging for practical PMSM drives. Experimental validation under abrupt load application and load rejection will also be carried out using a loading device capable of fast torque step changes. Online parameter identification, parameter compensation, dead-time compensation, and filtering methods will be considered to further improve the robustness and practical applicability of the proposed sensorless speed estimation strategy.

## Figures and Tables

**Table 1:** Simulation platform parameters.

Items	Values	Items	Values
Stator winding resistance $R_{s}$	0.51 Ω	Flux linkage $\psi_{f}$	0.00838 Wb
Stator winding inductance $L_{d}$	0.295 mH	Rotational inertia *J*	0.0028 kg·m^2^
Stator winding inductance $L_{q}$	0.295 mH	Viscous damping coefficient *B*	0.0002 N·m·s
Maximum speed $\omega_{\max}$	3000 r/min	Pole pairs $P_{n}$	4
DC-link voltage $V_{DC}$	24 V		

**Table 2:** Comparison of accuracy and computational cost between 9 and point enumeration and different ternary search iteration numbers.

Method	Iterations	Evaluations	Speed Error (r/min)	Settling Time (s)
Fixed-step 9-point enumeration	9	9	2.0	0.040
Ternary search	2	4	1.3	0.025
Ternary search	4	8	1.0	0.027
Ternary search	6	12	0.7	0.035

**Table 3:** Quantitative comparison of experimental speed estimation performance under different speed transitions.

(a) 500 r/min to 1000 r/min			
**Method**	**Settling Time (s)**	**Maximum Speed Error (r/min)**	**Steady-State Speed Error (r/min)**
Fixed-step FCS-MPC, 1°	0.3	136	20
Fixed-step FCS-MPC, 10°	0.2	164	80
Fixed-step FCS-MPC, 20°	0.1	190	86
Proposed method	Negligible	8	6
**(b) 1000 r/min to 1500 r/min**			
**Method**	**Settling Time (s)**	**Maximum Speed Error (r/min)**	**Steady-State Speed Error (r/min)**
Fixed-step FCS-MPC, 1°	0.20	120	40
Fixed-step FCS-MPC, 10°	0.05	136	96
Fixed-step FCS-MPC, 20°	0.03	140	106
Proposed method	Negligible	12	10

**Table 4:** Real-time computational performance comparison on the STM32F407 platform.

Method	Average Time (μs)	Maximum Time (μs)	CPU Load (%)	Flash Usage (Bytes)	RAM Usage (Bytes)
Fixed-step FCS-MPC	56	60	60	16,720	2592
Proposed method	46	57	57	16,748	2600

## Data Availability

Data are contained within the article.
